# Editorial: Improving the Nutritional Content and Quality of Crops: Promises, Achievements, and Future Challenges

**DOI:** 10.3389/fpls.2019.00738

**Published:** 2019-06-06

**Authors:** Felipe Klein Ricachenevsky, Marta Wilton Vasconcelos, Huixia Shou, Alexander Arthur Theodore Johnson, Raul Antonio Sperotto

**Affiliations:** ^1^Biology Department, Federal University of Santa Maria, Santa Maria, Brazil; ^2^Universidade Católica Portuguesa, CBQF - Centro de Biotecnologia e Química Fina - Laboratório Associado, Escola Superior de Biotecnologia, Porto, Portugal; ^3^The State Key Lab of Plant Physiology and Biochemistry, College of Life Sciences, Zhejiang University, Hangzhou, China; ^4^School of BioSciences, The University of Melbourne, Melbourne, VIC, Australia; ^5^Graduate Program in Biotechnology, University of Taquari Valley - Univates, Lajeado, Brazil

**Keywords:** nutritional quality, biofortification, plant nutrition, iron, zinc, transporter

Plants are the ultimate source of nutrients for humans and livestock. To date, we use only a handful of species for our subsistence and focus mostly on carbohydrate-rich grains such as wheat, corn and rice. However, these starch-rich cereals are typically nutrient-poor, leaving populations that base their diets on starchy grains with low intakes of many essential nutrients. At the same time, seeds, leaves and roots can accumulate toxic elements depending on species, genotype and growth conditions, representing potential hazards to human health. Improving the nutritional quality of plants, which includes both nutrient enrichment of edible tissues and minimizing the likelihood of contamination, has been a focus of research for years. In this Research Topic a diverse collection of reviews, opinions and original research articles highlight the achievements and future directions in this field.

## Biofortification/Nutritional Quality: an Overview

Garg et al. review a range of biofortification studies in different crops involving transgenic, conventional, and agronomic approaches and the use of biotechnology, crop breeding, and fertilization, respectively. The authors discuss the limitations of each approach and the challenges that transgenic biofortified crops face regarding consumer acceptance. Taking a different perspective, Capstaff and Miller review the current knowledge and future direction for yield and nutritional quality improvement of forage crops, which have received relatively limited attention compared to cereals, fruits, and vegetables. The authors also discuss the applicability of knowledge obtained from model plants and grain crops coupled with the availability of genomics and bioinformatics to generate improved forage crops for food security. The review by Hameed et al. presents an overview of nutritional improvement efforts in potato (*Solanum tuberosum*) using both traditional breeding and genetic engineering techniques, including nutrient concentration increase and anti-nutrient decrease. The authors also discuss constraints to potato production, such as biotic and abiotic stresses, post-harvest quality, unfavorable soil and climatic factors. Moreover, genetically modified potato risk assessment/regulation and future breeding techniques using TALENs and CRISPR/Cas9 are discussed.

Paul et al. review progress in the Banana21 biofortification program, which aims to increase pro-vitamin A content (and iron in the future) in cooking banana, which is a staple food in Uganda. Vitamin A deficiency is one of the most widespread nutritional problems worldwide, affecting millions of people, and is especially prevalent in children. Importantly, the program includes technology transfer and aims to produce and deregulate transgenic local varieties of banana. Authors provide an overview of the strategies used, to achieve the goal of delivering at least 50% of the estimated average requirement of pro-vitamin A in 300 g of banana per day.

Other reviews discuss biofortification for specific nutrients. Strobbe and Van Der Straeten focus on the role of thiamin (B1), pyridoxine (B6), and folates (B9) in plant physiology. Biofortification strategies to enhance B-vitamin in crops using metabolic engineering or breeding are presented. The authors also discuss the concept of multi-biofortification, the simultaneous biofortification of multiple vitamins and minerals, and the possible synergistic or adverse effects of such combinations. Lyons reviews the approaches used for agronomic biofortification of crops with iodine (I) and selenium (Se) in major crops, with a special focus on wheat. Although I is provided by salt iodization, this approach has been shown to be insufficient. The author shows problems derived from combined deficiency in both nutrients, and how current approaches to provide enough I and Se to humans are not sufficient. The review discusses approaches to I and Se agronomic biofortification, comparing results of soil and foliar application of I and Se fertilizers. Finally, Puranik et al. assess recent advances and challenges for Ca biofortification in finger millet (*Eleusine coracana*), a crop with inherently high grain Ca content, but which needs to be in a bioavailable form to impact human nutrition. As such, integration of a naturally Ca-rich crop like finger millet into global biofortification programs could be a good starting point to alleviate human Ca malnutrition. According to the authors, large-scale protein profiling to identify the complete set of proteins involved in finger millet Ca homeostasis is still unavailable.

López-Pérez et al. present a dynamic model to describe absorption, transport, and deposition rates of silicon (Si) in tomato. The model consists of six state equations, using as inputs key environmental factors related to Si absorption and mobilization, such as temperature, pH, CO_2_ concentration, and soil organic matter. Use of the model can increase understanding of the agronomic management of Si in plants. On a similar line, da Rosa Couto et al. explore the plants grown in soils with long-term animal waste applications as fertilizers and the potential grain contamination with heavy metals. Although important for both traditional and organic farming, the recurrent use of animal waste can result in changes to soil chemistry that make plants more prone to accumulation of heavy metals, which can harm consumers. The manuscript estimates the potential contamination risks associated with soil concentration of heavy metals after organic waste usage reported in the literature, and calls attention to the need for more careful monitoring to understand how different plant species and their edible parts can become a source of dietary contamination for humans.

## Reviews on Fe and Zn Biofortification

Nutritional quality includes many topics, but certainly iron (Fe) and zinc (Zn) biofortification has been one of the most prolific areas in the field. Here we feature updated reviews and opinions focused on these two essential micronutrients that are commonly lacking in human diets. Garcia-Oliveira et al. review the genetic basis of Fe and Zn accumulation in cereals and describe how modern breeding technologies are helping to promote essential element accumulation and bioavailability, while minimizing the accumulation of anti-nutrients such as phytate and hazardous heavy metals that often are transported along with Fe and Zn. The authors discuss how existing genetic variability can contribute to breeding of biofortified cereals, and some of the methodological difficulties in reliably measuring Fe and Zn. Sperotto and Ricachenevsky discuss common bean (*Phaseolus vulgaris*) biofortification efforts, and how model species lessons can provide shortcuts in finding pathways and candidate genes for Fe, Zn and anti-nutrient manipulation in beans. In particular, the possibility of tissue-specific biofortification of common bean seed coat and cotyledons, as well as decreasing phytate and polyphenols anti-nutrients in the same tissues, is discussed.

The mini-review by Ricachenevsky et al. describes the role of root vacuoles in controlling symplastic concentrations of nutrients and toxic trace elements, which in turn affects shoot accumulation. Examples from the literature regarding natural variation in vacuolar transporters and loss-of-function mutants show that the more abundantly an element is stored in root vacuoles, the less it is loaded into the xylem and translocated to shoots and seeds. Thus, manipulation of root storage capacity should be considered in biofortification approaches. In another mini-review, Nozoye highlights the usefulness of increasing nicotianamine (NA) levels in plants, which is achieved by over-expressing nicotianamine synthase (NAS) genes. NA is a metal chelator involved in metal translocation. Plants that accumulate more NA increase Fe and Zn concentrations in edible tissues and can also become more tolerant to Fe deficiency.

## Experimental Approaches to Improve Nutritional Quality

Manuscripts in this section describe experimental methodologies important for the improvement of nutritional quality in plants. Due to the high bioavailability of rice seed storage protein (SSP) for human and animal nutrition, increased SSP content is one of the main breeding objectives for improving the nutritional quality of rice. Chen et al. investigate albumin, globulin, prolamin, glutelin, and total SSP contents in milled rice of 527 rice accessions grown in two environments. By associating these nutrient traits with genome sequencing data, they identify novel SNPs and candidate genes related to rice seed protein content and composition which may be useful for future rice molecular breeding strategies aiming at quality improvement.

It is well-known that daily consumption of fruits and vegetables lowers the risk of several chronic illnesses, including cardiovascular disease, diabetes, and cancer. The health benefits of vegetable and fruit crops are often attributed to their high content of specific health promoting compounds such as fibers, polyphenols, and vitamins. Glycine is the most abundant free amino acid in horticultural soils and Yang et al. show that exogenous glycine supplementation can increase the accumulation of health-promoting compounds and enhance antioxidant activity in hydroponically grown lettuce.

Starch is the main carbohydrate form in wheat grain. Other than the major components of amylose and amylopectin, starch can also interact with minor components such as lipids, proteins, and phosphorus. It is understandable that phosphorus supplies must affect the yield and quality of wheat grain. Zhang et al. employ three levels of phosphorus fertilizer application to wheat fields and find that different levels of phosphorus significantly influence the expression of starch biosynthesis genes, starch synthesis, degradation, and microstructure in wheat grains. The results provide knowledge about the importance of applying appropriate amounts of phosphorus fertilizers for the improvement of wheat yield and starch quality.

The high affinity nitrate transporter OsNRT2.1 plays a role in nitrogen uptake and translocation in rice. Luo et al. show that OsNRT2.1 overexpression increases not only nitrate uptake, but also Mn accumulation in rice grains. The reason for high accumulation of Mn by OsNRT2.1 overexpression is probably due to elevated expression of Mn transporter genes, including OsNRAMP3, 5, and 6. This work provides an alternative approach for increasing Mn uptake in plants and could have implications for grain quality.

## Experimental Approaches to Fe and Zn Biofortification

We highlight a series of original research papers focused on increasing Fe and Zn accumulation in crops. Díaz-Benito investigate wild-type rice and six transgenic rice lines overexpressing nicotianamine synthase (*OsNAS1*) and/or barley nicotianamine amino transferase (*HvNAATb*) in order to elucidate the role of 2-deoxymugineic acid (DMA) and nicotianamine (NA) on metal distribution in the rice embryo and endosperm. Using a series of approaches the authors conclude that when there are increases in DMA concentration alone or in combination with NA, the prevalent mechanism of seed Fe loading is via Fe(III)-DMA. The study also highlights that a better understanding of transgenic plant phenotypes, using in-depth localized quantification of targeted nutrients, will improve the efficacy of future biofortification strategies. In a similar topic, Tan et al. reports on a strategy for Fe biofortification of chickpea (*Cicer arietinum*) using genetic engineering. The authors successfully transformed cultivar HatTrick with chickpea nicotianamine synthase 2 (*CaNAS2*) and soybean (*Glycine max*) ferritin (*GmFER*) constitutive expression cassettes. Analysis of NA and Fe levels in the transformed seeds revealed that NA levels were twice those of control lines. The authors suggest that this may have important ramifications in terms of increasing Fe bioavailability in chickpea grains.

Zhang et al. examine two sets of backcrossed inbred lines derived from the same donor, and two recipient elite varieties from Southwestern China, to determine the effect of genetic background and environment on grain mineral concentration by QTL mapping. This study allowed confirmation of the results of a genome-wide association study (GWAS) using a set of 698 sequenced accessions, and favorable haplotypes of Fe, Zn, Cd, Mn, Cu, and Se candidate genes were identified. In particular, 37 genes (19.3%) were found to be significantly associated between the QTL targeting traits and the haplotype variations by pair-wise comparison, and these genes may be useful for future rice biofortification strategies.

Singh et al. tackle the challenge of increasing Zn, Fe and protein concentration in wheat grain by applying exogenous nitrogen (N), Fe, and Zn at different rates and application times. Apart from increased grain yield, relatively higher protein content and Fe/Zn concentration were recorded in wheat grain when a split N application was applied. Furthermore, soil and foliar Fe/Zn supplies combined with a single application of N at sowing increased Zn and Fe concentrations by 46% and 35%, respectively, relative to controls. These results indicate that proper management of N, Fe, and Zn application may could enhance grain protein content and Fe/Zn concentration in wheat.

## Fe and Zn Physiology Studies to Provide Tools for Biofortification

Aiming to develop a new method to quickly characterize rice genes related to metal homeostasis (in particular Fe and Zn), Ricachenevsky et al. investigate the ionome of Arabidopsis Full Length Over-eXpressor (FOX) lines with heterologous expression of rice cDNAs driven by the 35S promoter. The authors identified two lines overexpressing *OsZIP7*, which had 25% increase in shoot Zn concentrations compared to control. Ricachenevsky et al. found that the gene was able to complement a Zn transport defective yeast mutant, the protein is localized to the plasma membrane, and the seeds of the overexpressing lines had significantly higher Zn concentrations. This technique shows promise toward identifying candidate genes for mineral enhancement.

Phytoremediation has frequently been defined as a sustainable bioremediation process that uses various types of plants to remove, transfer, stabilize, and/or destroy heavy metals from the environment in which they grow. In this context, it is important to choose the best “crop for the job”. Papiernak et al. suggest tobacco as a possible crop to fulfill this goal. However, despite the interest in the use of tobacco to remove metals from contaminated soil, knowledge of the processes by which tobacco exerts this role is still limited. The authors strived to identify new Zn transport genes in tobacco using an *in silico* approach, gene expression data, gene cloning and functional analysis in yeast (heterologous expression). The authors conclude that NtZIP1- like is localized at the plasma membrane, in the roots and shoots, and is involved in Zn transport. NtZIP1-like appears responsible for Zn uptake by root cells of the mature basal zone and may be involved in a mechanism to protect the root and leaf cells from accumulating excess Zn.

Chan-Rodriguez and Walker perform a genetic dissection of yellow-stripe mutants available in the Maize Genetic Cooperation Stock Center (MGCSC). Grasses are known to rely on Fe(III) chelation for Fe uptake, which involves phytosiderophore secretion and Fe(III)-phytosiderophore complex transport into root cells. Yellow-stripe phenotypes are indicative of Fe deficiency in leaves, and one of the first mutants characterized in maize (*ys1*) contained mutations in the Yellow-Stripe 1 (YS1) transporter, which is the Fe(III)-phytosiderophore transporter (Curie et al., 2001), while another (*ys3*) is suggested to have mutations in the phytosiderophore secretion transporter TOM1 (Nozoye et al., 2013). Authors screen 31 yellow-stripe mutants from maize, identifying some allelic to *ys1* and *ys3*, as well as three new, non-allelic mutants which have low Fe levels in shoots and may represent new players in Fe uptake mechanisms of grasses.

Abdallah et al. describe a non-model plant species (*Hedysarum carnosum*), from semi-arid areas in Tunisia, where soils are saline-alkaline and present low Fe availability. Three isolates from *H. carnosum* were characterized for morphological and physiological responses to low Fe conditions, showing rhizosphere acidification and IRT1-ortholog upregulation, but no Fe reductase activity in roots. Interestingly, one ecotype showed increased Fe deficiency tolerance, which may be linked to more pronounced IRT1 expression under control conditions. This study lays the foundation for using *H. carnosum* to study adaptation to extreme conditions.

## Gene Family Characterization

Two studies provide basic knowledge on important transporter gene families which could be important for further biofortification and/or plant nutrition studies. Qin et al. identify 13 NRAMP genes in the soybean genome and document variable expression profiles of *GmNRAMP* genes among tissues and in response to nutrient stress, suggesting that GmNRAMP proteins perform a range of functions in specific tissues throughout development. Subcellular localization analysis in Arabidopsis protoplasts confirm the tonoplast or plasma membrane localization of these proteins. Teng et al. conduct a genome-wide sequence analysis of *PHT1* (phosphate (Pi) transporters) genes in wheat and clone 21 of the genes. The cloned *PHT1* genes show Pi-transport activity in yeast cells grown under both low and high Pi conditions. Expression of some *TaPHT1* genes at flowering stage positively correlated with P uptake after stem elongation across three P application rates and two wheat varieties, suggesting that modification in *PHT1* gene expression may improve P use efficiency under a wide range of P concentrations.

## Final Comment

This Research Topic provides readers with a wide range of manuscripts in plant nutrition and describes novel results relevant to food security and global health.

## Author Contributions

All authors listed have made a substantial, direct and intellectual contribution to the work, and approved it for publication.

### Conflict of Interest Statement

The authors declare that the research was conducted in the absence of any commercial or financial relationships that could be construed as a potential conflict of interest.
